# The Japanese herbal medicine Hangeshashinto enhances oral keratinocyte migration to facilitate healing of chemotherapy-induced oral ulcerative mucositis

**DOI:** 10.1038/s41598-019-57192-2

**Published:** 2020-01-17

**Authors:** Kanako Miyano, Moeko Eto, Suzuro Hitomi, Takashi Matsumoto, Seiya Hasegawa, Ayane Hirano, Kaori Nagabuchi, Noriho Asai, Miaki Uzu, Miki Nonaka, Yuji Omiya, Atsushi Kaneko, Kentaro Ono, Hideaki Fujii, Yoshikazu Higami, Toru Kono, Yasuhito Uezono

**Affiliations:** 1 Division of Cancer Pathophysiology, National Cancer Center Research Institute, Tokyo, 104-0045 Japan; 2 Laboratory of Molecular Pathology and Metabolic Disease, Faculty of Pharmaceutical Sciences, Tokyo University of Science, Chiba, 278-8510 Japan; 3 Division of Physiology, Kyushu Dental University, Fukuoka, 803-8580 Japan; 4 Tsumura Kampo Research Laboratories, Tsumura & Co., Ibaraki, 300-1192 Japan; 5 Department of Medicinal Chemistry, School of Pharmacy, Kitasato University, Tokyo, 108-8641 Japan; 6 Translational Research Center, Research Institute of Science and Technology, Tokyo University of Science, Chiba, 278-8510 Japan; 7 Center for Clinical and Biomedical Research, Sapporo Higashi Tokushukai Hospital, Sapporo, Hokkaido Japan; 8 Division of Supportive Care Research, Exploratory Oncology Research and Clinical Trial Center, National Cancer Center, Tokyo, 104-0045 Japan; 9 Innovation Center for Supportive, Palliative and Psychosocial Care, National Cancer Center Hospital, Tokyo, 104-0045 Japan

**Keywords:** Chemotaxis, Receptor pharmacology

## Abstract

Chemotherapy often induces oral ulcerative mucositis (OUM) in patients with cancer, characterized by severe painful inflammation. Mouth-washing with the Japanese herbal medicine hangeshashinto (HST) ameliorates chemotherapy-induced OUM in patients with colorectal cancer. Previously, we demonstrated that HST decreased interleukin 1β-induced prostaglandin E2 production in human oral keratinocytes (HOKs) and OUM-induced mechanical or spontaneous pain in rats. However, HST effects on tissue repair functions in HOKs remain unclear. Here, we examined the effects of HST on scratch-induced wound healing *in vitro* and *in vivo*. *In vitro*, HST enhanced wound healing mainly through scratch-induced HOK migration. Screening of the seven constituent medicinal herbs and their major components revealed that Scutellaria root, processed ginger, and Glycyrrhiza components mainly induced the scratch-induced HOK migration. Pharmacokinetic analyses indicated that the active ingredient concentrations in rat plasma following oral HST administration were below the effective doses for HOK migration, suggesting direct effects of HST in OUM. Mitogen-activated protein kinase and C-X-C chemokine receptor 4 inhibitors significantly suppressed HST-induced HOK migration. Moreover, HST enhanced tissue repair in our OUM rat model. Thus, HST likely enhanced OUM tissue repair through oral keratinocyte migration upon MAPK and CXCR4 activation and may be useful in patients with cancer-associated OUM.

## Introduction

Patients with cancer receiving chemotherapy, radiotherapy, hematopoietic stem cell transplant, and terminal care often present with severe oral ulcerative mucositis (OUM), which evokes painful inflammation and limits fundamental life behaviours such as ‘eating, drinking, and talking’^1–15^. In addition, OUM increases the risk of systemic infection via opportunistic microorganisms, which may lead to the extension of hospitalization^5,15–19^. OUM also often induces patients with cancer to discontinue/modify their therapy regimen, which adversely affects patient prognosis^13,15,16,20^. Therefore, the effective management of OUM is indispensable for improving both the quality of life and prognosis for the patient^15^.

Hangeshashinto (HST), a traditional Japanese medicine (Kampo medicine), contains the extracted ingredients of seven medicinal herbs and has been approved by Japan’s Ministry of Health, Labour and Welfare as a prescribed medicine^15,21^. From the 16th century to the present, HST has been used in Japan to treat inflammatory diarrhoea, gastritis, and oral mucositis. Recently, double-blind, placebo-controlled, randomized studies have reported that repetitive mouth-washing with HST was effective for the improvement of chemotherapy-induced OUM in patients with colorectal or gastric cancer^22^. Several *in vitro* studies also reported that HST induced antibacterial and antioxidant effects, suggesting that these effects were involved in the mechanisms of HST with respect to the improvement of OUM^23–25^. Moreover, our previous studies *in vitro* and *in vivo* revealed additional mechanisms of HST in OUM, including both anti-inflammatory and analgesic effects^26–28^. Nevertheless, an important process during the restoration of ulcer sites is rapid repair of the ulcer site by migration and covering of the epithelium cells. However, the effects of HST on the wound-induced migration of oral keratinocytes or OUM healing (tissue repair) remain unclear.

In the present study, we therefore examined the effects of HST on wound healing using both *in vitro* and *vivo* experiments. First, we analysed the effects of HST on scratch-induced wound healing using human oral keratinocytes (HOKs) and identified the active medicinal herbs in HST and their ingredients by screening the seven constituents and their major components. Second, to reveal the actions of these active ingredients via the blood, we evaluated their pharmacokinetics rat plasma along with the effects of several inhibitors, such as against intracellular signalling molecules, on HST-mediated scratch-induced wound healing. Finally, we examined the effects of HST on OUM healing using an OUM rat model.

## Results

### HST enhances scratch-induced wound healing mainly by facilitating HOK migration *in vitro*

**Figure 1:**
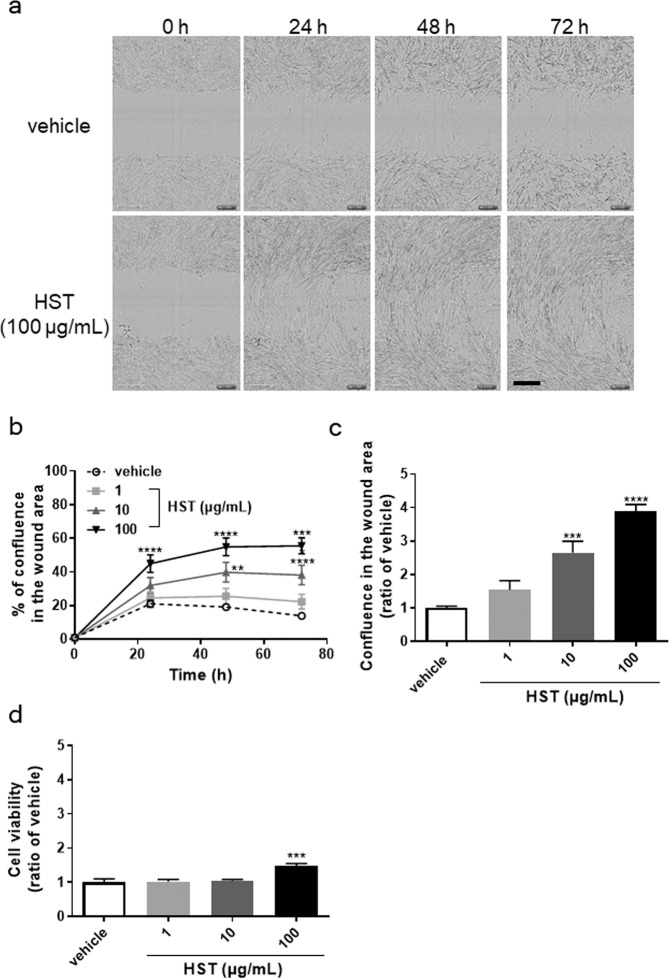
Effects of HST on the scratch-induced migration and cell viability of HOKs. Scratch-induced migration was measured using the IncuCyte ZOOM system. (**a**) Images showing the scratched area in HOKs treated with vehicle or HST (10, 100 µg/mL) for 0, 24, 48, and 72 h. (**b**) Effect of HST (1, 10, 100 µg/mL) on the scratch-induced migration of HOKs over time. (**c**) Extent of the scratch-induced migration of HOKs treated with HST (1, 10, 100 µg/mL) for 72 h calculated as the ratio of vehicle [confluence of HOKs (%) in wound area treated with HST/that treated with vehicle]. (**d**) Cell viability in HOKs treated with vehicle or HST (1, 10, 100 µg/mL) for 72 h. Data are expressed as the ratio of vehicle at 72 h. **p < 0.01, ***p < 0.001, and ****p < 0.0001, compared with vehicle; Tukey’s multiple comparisons test following one- (**c**) or two-way (**b**) ANOVA.

We first examined the effects of HST on scratch-induced wound healing using HOKs *in vitro*. Compared with the vehicle, HOKs treated with HST (100 µg/mL) covered the wound area in a time-dependent manner (Fig. 1a and Supplementary Movie S1). The extent of the wound healing area was then calculated as the percentage of confluence of HOKs in the wound area (Fig. 1b). HST significantly promoted scratch-induced wound healing in a time and a dose-dependent manner (Fig. 1b). Treatment with 10 and 100 µg/mL HST for 72 h significantly enhanced the scratch-induced wound healing (Fig. 1c), with the effect of 100 µg/mL HST being approximately 4-fold higher than that of the vehicle [Fig. 1c; vehicle vs. 100 µg/mL HST (mean ± S.E.M.); 1.0 ± 0.06 vs. 3.89 ± 0.20].

In addition, we investigated the effects of HST on cell growth in HOKs. HST at a concentration of 100 µg/mL but not 10 µg/mL significantly increased the cell growth (Fig. 1d), with this effect being approximately 1.5-fold higher than that of the vehicle [Fig. 1d; vehicle vs. 100 µg/mL HST (mean ± S.E.M.): 1.0 ± 0.10 vs. 1.65 ± 0.09], whose growth action was relatively smaller than action of wound healing. In addition, animations of the scratched area in HOKs treated with vehicle or 100 µg/mL HST also revealed that HOKs treated with HST covered the wound area via HOK migration (Supplementary Fig. S1). Taken together, these data suggested that HST enhanced the scratch-induced wound healing mainly by facilitating the migration of HOKs.

### Components of scutellaria root, processed ginger, and Glycyrrhiza enhance scratch-induced HOK migration *in vitro*

**Figure 2:**
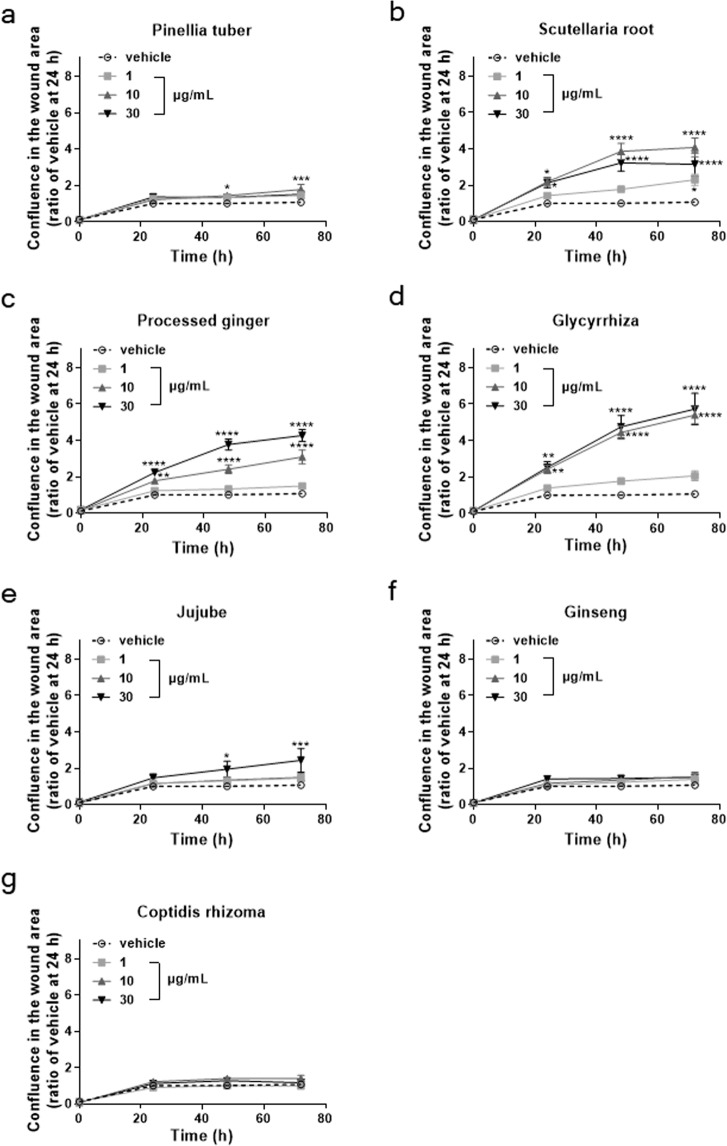
Among the seven medicinal herbs in HST, Scutellaria root, processed ginger, and Glycyrrhiza exhibited marked HOK migration effects. HOKs were treated with 1, 10, or 30 µg/mL of Pinellia tuber (**a**), Scutellaria root (**b**), processed ginger (**c**), Glycyrrhiza (**d**), jujube (**e**), ginseng (**f**), or Coptis rhizome (**g**). Data are expressed as the ratio of vehicle at 72 h. *p < 0.05, **p < 0.01, ***p < 0.001, and ****p < 0.0001, compared with vehicle; Tukey’s multiple comparisons test following two-way ANOVA.

HST contains seven medicinal herbs in the ratio Pinellia tuber (5):Scutellaria root (2.5):processed ginger (2.5):Glycyrrhiza (2.5):jujube (2.5):ginseng (2.5):Coptis rhizome (1). Thus, even Pinellia tuber constitutes at most 30% of HST. We therefore analysed the effects of 1, 10, or 30 µg/mL of the seven individual medicinal herbs on scratch-induced HOK migration to reveal which medicinal herbs in HST were involved in facilitating scratch-induced wound healing. As shown in Fig. 2, Scutellaria root, processed ginger, and Glycyrrhiza markedly facilitated the scratch-induced HOK migration in a dose-dependent manner. Pinellia tuber and jujube also significantly induced HOK migration, although to a lesser degree. Neither ginseng nor Coptis rhizome affected the scratch-induced migration (Fig. 2). Therefore, Scutellaria root, processed ginger, and Glycyrrhiza were used in subsequent analyses.

To clarify which components in the three identified active herbs enhanced the scratch-induced HOK migration, we screened their ingredients using a wound healing assay. Among the ingredients of Scutellaria root (baicalin, baicalein, and wogonin), only baicalein at 10 µM significantly enhanced scratch-induced HOK migration, whereas all components of processed ginger ([6]-shogaol, [8]-shogaol, [10]-shogaol, [6]-gingerol, [8]-gingerol, and [10]-gingerol) showed a significant effect (Table 1). In comparison, among Glycyrrhiza components (glycyrrhizin, glycyrrhetinic acid, liquiritin, isoliquiritin, liquiritin apioside, and isoliquiritigenin), only glycyrrhetinic acid (10 µM) significantly enhanced scratch-induced HOK migration (Table 1).

**Table 1:** Effects of components of Scutellaria root, processed ginger, and Glycyrrhiza on the scratch-induced migration of HOKs.

Medicinal herb	Ingredients	Confluence (ratio of vehicle)		
		0.1 μM	1 μM	10 μM
Scutellaria root	baicalin	−	1.71 ± 0.14	2.51 ± 0.53
	baicalein	−	1.70 ± 0.27	3.02 ± 0.76**
	wogonin	−	1.62 ± 0.21	0.98 ± 0.15
Processed ginger	[6]-shogaol	2.76 ± 0.46*	3.49 ± 0.55***	0.99 ± 0.26
	[8]-shogaol	2.33 ± 0.44	2.84 ± 0.43**	3.77 ± 0.48****
	[10]-shogaol	1.91 ± 0.31	3.24 ± 0.41***	3.48 ± 0.37****
	[6]-gingerol	−	2.12 ± 0.20	3.48 ± 0.30****
	[8]-gingerol	−	2.02 ± 0.21	2.95 ± 0.47**
	[10]-gingerol	2.20 ± 0.30	3.02 ± 0.38**	2.21 ± 0.21
Glycyrrhiza	Glycyrrhizin	−	1.05 ± 0.14	1.58 ± 0.33
	Glycyrrhetinic acid	−	1.20 ± 0.11	3.31 ± 0.85****
	Liquiritin	−	1.50 ± 0.08	1.69 ± 0.13
	Isoliquiritin	−	1.34 ± 0.12	1.51 ± 0.20
	Liquiritin apioside	−	1.27 ± 0.20	1.53 ± 0.33
	Liquiritigenin	−	1.34 ± 0.21	1.53 ± 0.08
	Isoliquiritigenin	−	1.75 ± 0.16	1.55 ± 0.16

HOKs were treated with various ingredients of Scutellaria root (baicalin, baicalein, and wogonin), processed ginger ([6]-shogaol, [8]-shogaol, [10]-shogaol, [6]-gingerol, [8]-gingerol, and [10]-gingerol), and Gglycyrrhiza (glycyrrhizin, liquiritin, isoliquiritin, liquiritin apioside, and isoliquiritigenin) for 72 h. Data are expressed as the ratio of vehicle at 72 hr. *p < 0.05, **p < 0.01, ***p < 0.001, and ****p < 0.0001, compared with vehicle; Tukey’s multiple comparisons test following one-way ANOVA.

### Plasma concentrations of compounds derived from Scutellaria root, processed ginger, and Glycyrrhiza in rats

**Figure 3:**
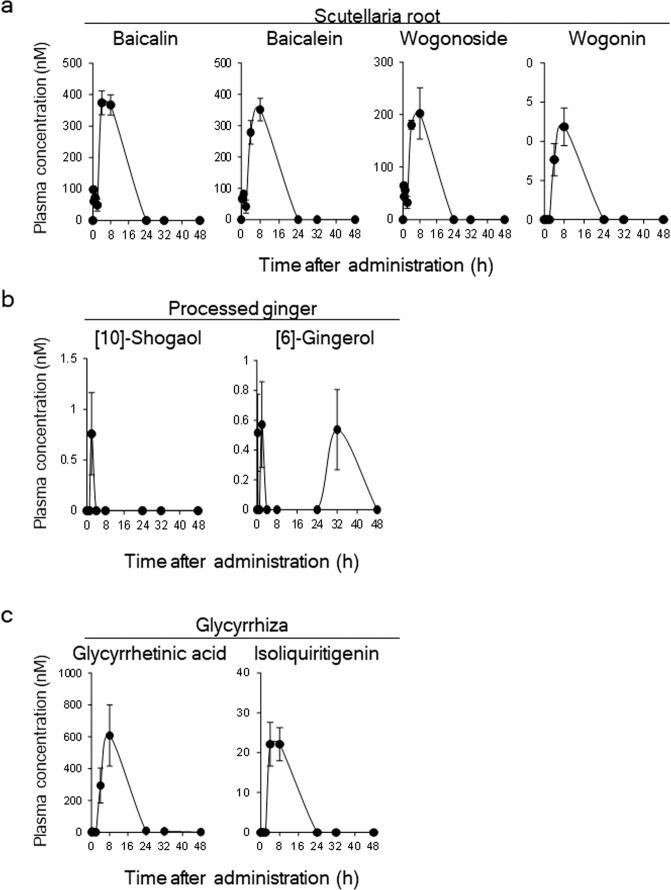
Plasma concentration of ingredients following oral administration of HST. Plasma samples were collected at 0 (pre-administration), 0.25, 0.5, 1, 2, 4, 8, 24, 32, and 48 h following HST administration. Measurement for ingredient concentration was performed via liquid chromatography-tandem mass spectrometry.

To clarify the actions of HST on scratch-induced HOK migration via the blood, we first examined plasma concentrations of the HST medicinal herb components identified as showing activity *in vitro*. Among the 12 compounds analysed in this study (Table 2), 8 were detected in the plasma following oral administration of HST (Fig. 3). The maximum concentration of glycyrrhetinic acid was highest with 607.6 nM, followed by baicalin (374.1 nM) and baicalein (351.2 nM). The plasma concentration-time curves of several detected compounds reached a peak at approximately 8 h following HST administration. The plasma concentrations of [6]-shogaol, [8]-shogaol, [8]-gingerol, and [10]-gingerol were below the limit of quantification.

**Table 2:** List of HST components analysed in the pharmacokinetic study.

Medicinal herb	Ingredients
Scutellaria root	baicalin
	baicalein (glucuronide of baicalein)
	wogonin
	wogonoside (glucuronide of wogonin)
Processed ginger	[6]-shogaol
	[8]-shogaol
	[10]-shogaol
	[6]-gingerol
	[8]-gingerol
	[10]-gingerol
Glycyrrhiza	Glycyrrhetinic acid
	Isoliquiritigenin

### HST enhances scratch-induced migration in HOKs via mitogen-activated protein kinases (MAPKs) and C-X-C chemokine receptor 4 (CXCR4)

**Figure 4:**
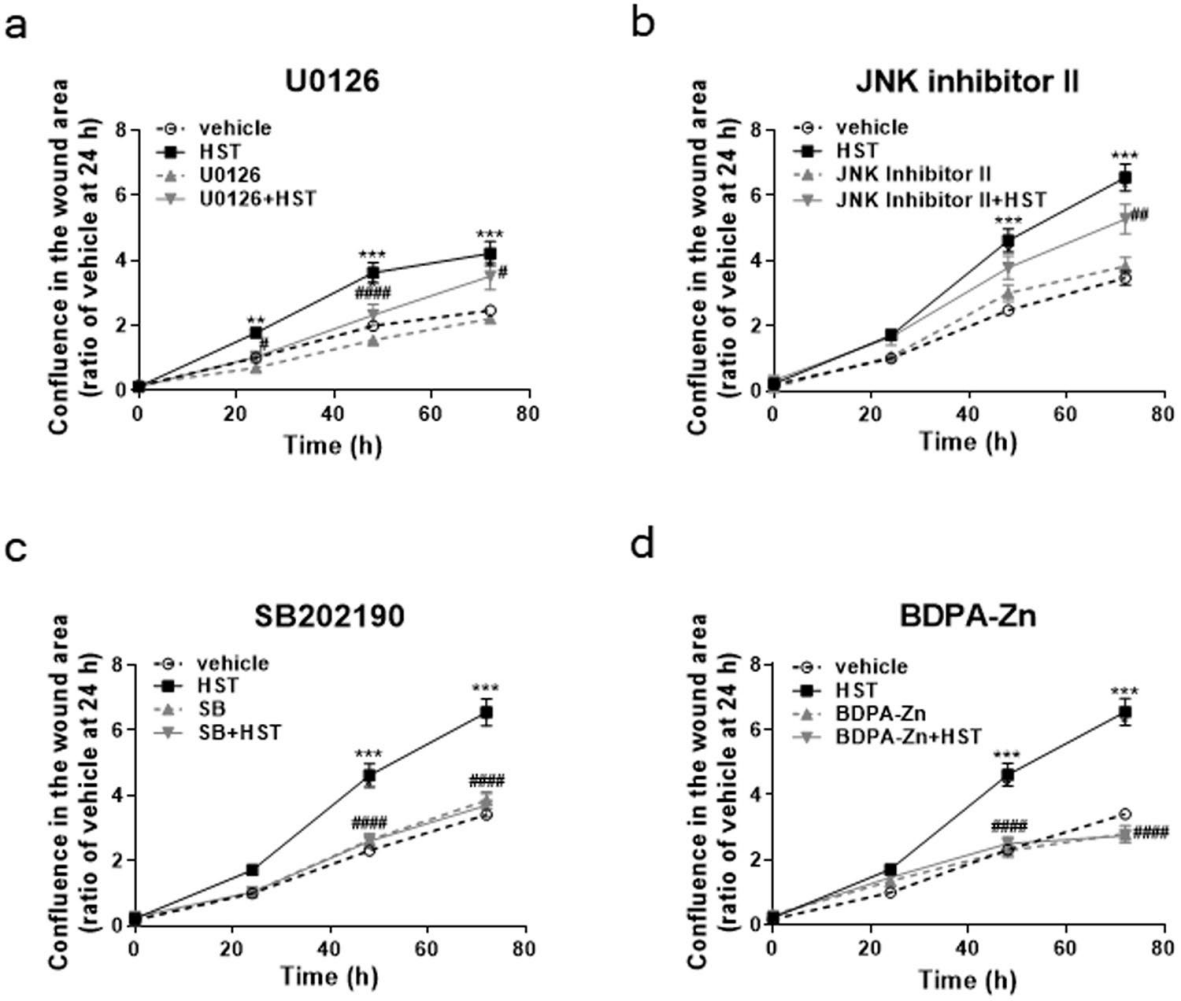
Effects of inhibitors of MAPKs or CXCR4 on the HST-induced enhancement of migration in HOKs. HOKs were co-treated with HST (100 µg/mL) and an ERK inhibitor U0126 (10 µM, **a**), JNK inhibitor II (1 µM, **b**), p38 inhibitor SB202190 (10 µM, **c**), or a CXCR4 inhibitor BDPA-Zn (3 µM, **d**). Data are expressed as the ratio of vehicle at 24 h. **p* < *0.01, and ****p < 0.0001, compared with vehicle; ^#^p* < *0.05, ^##^p < 0.01, and ^####^p < 0.0001, compared with HST: Tukey’s multiple comparisons test following two-way ANOVA.

Numerous reports have shown that MAPK and CXCR play important roles for cell migration^29–42^. To reveal the intracellular signalling mechanism of HST during scratch-induced HOK migration, we investigated the effects of inhibitors of MAPKs (extracellular signal-regulated kinase (ERK), c-Jun N-terminal kinase (JNK), and p38) and of CXCR4, which constitutes a crucial mediator of cell migration, on scratch-induced HOK migration. As shown Fig. 4a, the ERK inhibitor U0126 (10 µM) significantly decreased the HST-induced HOK migration at 24, 48, and 72 h. In addition, the p38 inhibitor SB202190 (10 µM) and the CXCR4 inhibitor BDPA-Zn (3 µM) completely suppressed the HST-induced HOK migration at 48 and 72 h (Fig. 4c, d). In contrast, the JNK inhibitor II (1 µM) partially suppressed the HST-induced HOK migration only at 72 h (Fig. 4b). These data suggested that MAPKs and CXCR4 are involved in HST-induced HOK migration.

### HST mitigates the severity of OUM development

**Figure 5:**
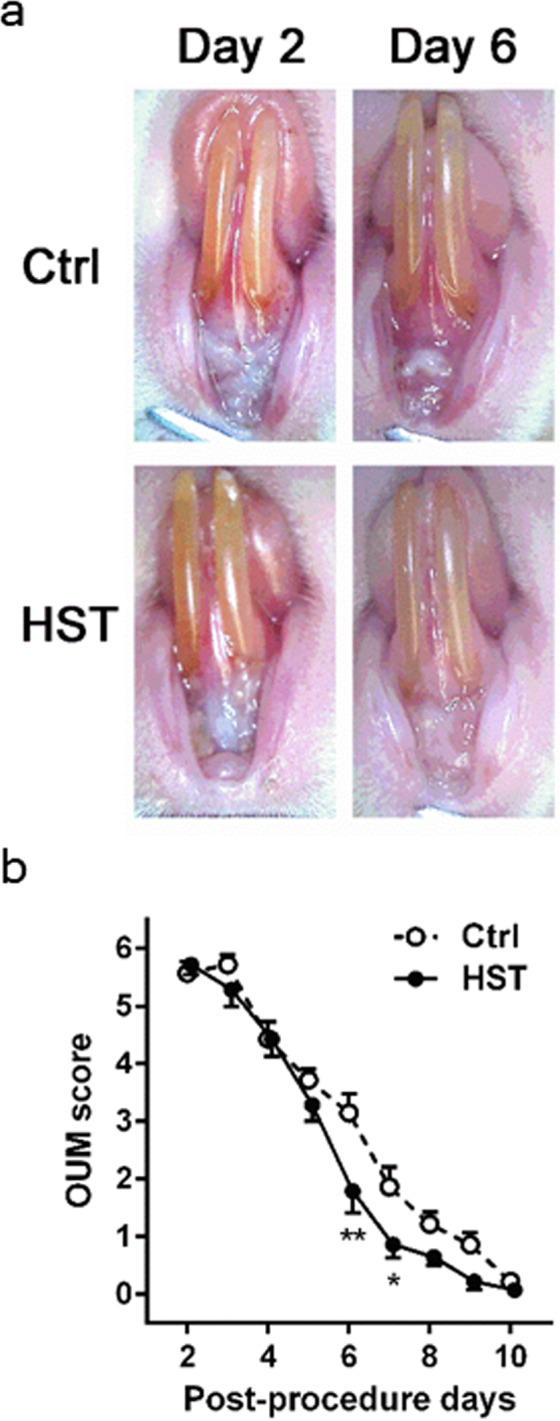
Effects of HST on oral ulcerative mucositis (OUM) healing. HST, rats administered a powder diet containing HST at a concentration of 1%; Ctrl, rats administered a normal powder diet. (**a**) Representative photographs of the ulcerative region in Ctrl and HST rats on days 2 and 6 following acetic acid treatment. (**b**) Daily changes of OUM scores following acetic acid treatment in Ctrl and HST rats (*n* = 7 in each group). *p* < *0.05 and **p < 0.01, compared with Ctrl rats; Sidak’s multiple comparisons test following two-way ANOVA.

No difference in body weight was observed between the control and HST-administered rats prior to and following 5-fluorouracil administration (before acetic acid treatment) (HST, 250.9 ± 9.3 g; control, 246.1 ± 5.3 g, *n* = 7 in each group). On day 6 after acetic acid treatment, the body weight was significantly increased in HST rats compared with that in control rats (HST, 274.3 ± 7.7 g; control, 260.7 ± 6.7 g). Ulcerations developed on day 2 after acetic acid treatment in both control and HST rats. The OUM score of HST-administered rats was significantly lower than that of control rats on day 6 and 7 following acetic acid treatment (Fig. 5).

### HST does not enhance the cell growth or scratch-induced migration of cancer cells *in vitro*

**Figure 6:**
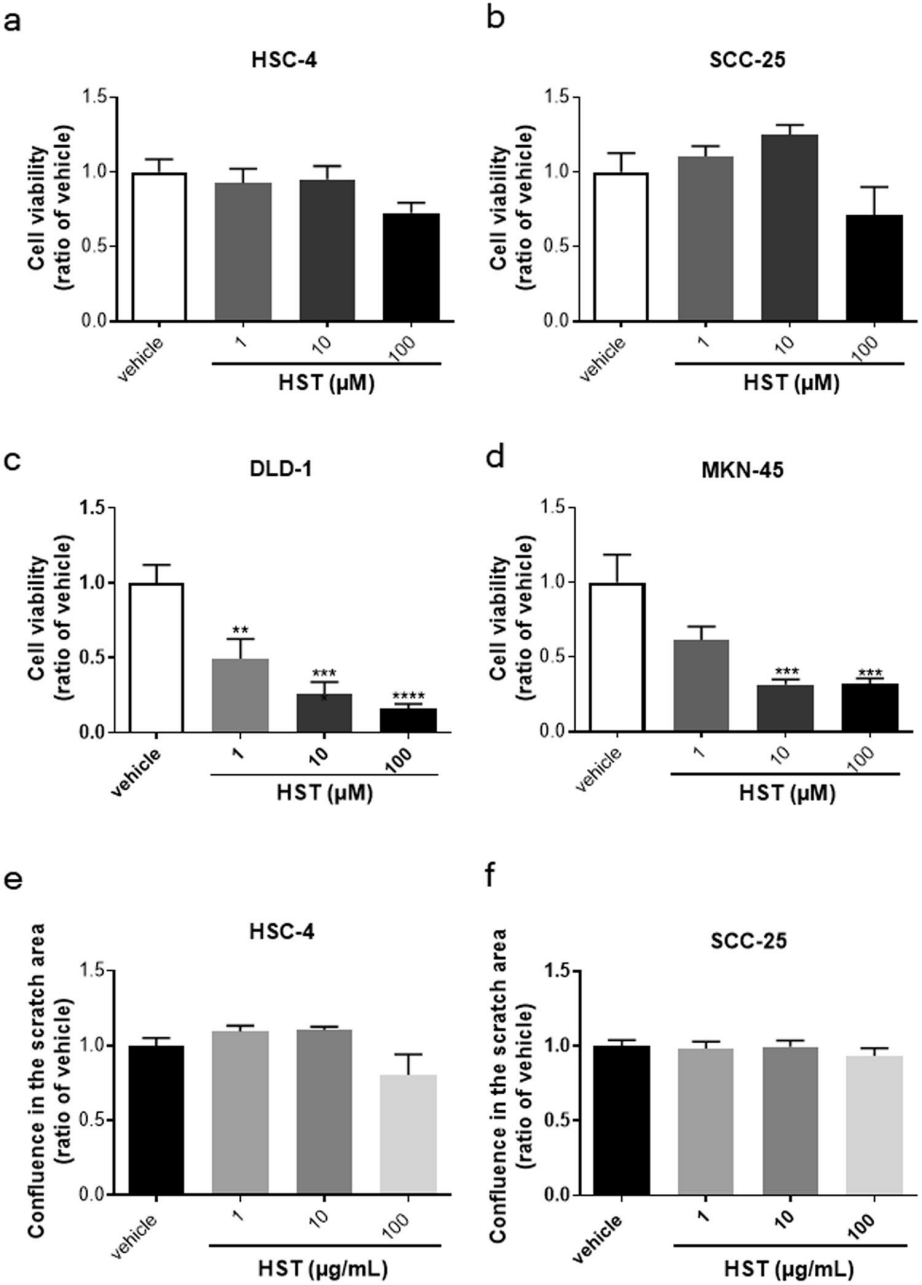
Effects of HST on the growth or scratch-induced migration of cancer cells *in vitro*. HSC-4 (**a**), SCC-25 (**b**), DLD-1 (**c**), and MKN-45 (**d**) cell lines were treated with HST for 72 h, then cell growth was measured using Cell Counting Kit-8. HSC-4 (**e**) and SCC-25 (**f**) cells were treated with HST (1, 10, and 100 µg/mL) for 72 h, then the area occupied by cancer cells on the scratched area was quantified using IncyCyte scratch wound cell migration software (ESSEN BioScience). Data are expressed as the ratio of vehicle at 72 h. *, **, ***, and **** indicate p < 0.05, 0.01, 0.001, and 0.0001, respectively, compared with vehicle; Tukey’s multiple comparisons test following one-way ANOVA.

To determine whether the stimulatory effects of HST were specific to normal (i.e., HOK) cells, we examined the effects of HST on cancer cell growth using cell lines derived from human squamous cell carcinoma of the tongue (HSC-4, SCC-25), colorectal cancer (DLD-1), and human gastric cancer (MKN-45). As shown in Fig. 6a and b, HST (1, 10, and 100 µg/mL) did not significantly enhance the cell viability of HSC-4 or SCC-25 cells, compared with the effects of vehicle treatment. In contrast, HST significantly decreased the cell growth of DLD-1 and MKN-45 cell lines (Fig. 6c, d).

In addition, we investigated the effects of HST on scratch-induced wound healing using HSC-4 and SCC-25 cell lines *in vitro*. Compared with the effects of vehicle treatment, HST (1, 10, and 100 µg/mL) did not significantly enhance the scratch-induced cell migration of these cell lines (Fig. 6e, f).

## Discussion

Our present study, for the first time, revealed that HST enhances scratch-induced wound healing. Although HST also enhanced the growth of HOKs, this effect was smaller than the wound healing effect (Fig. 1). In addition, the Supplementary Movie. S1 showed that the scratch-induced wound healing was induced by HOK migration. Taken together, these findings suggested that the effect of HST on scratch-induced wound healing might be due to migration rather than growth of HOKs. Moreover, we identified Scutellaria root, processed ginger, and Glycyrrhiza as active constituents among the seven medicinal herbs comprising HST (Fig. 2). We also found that the active ingredients of these herbs consisted of baicalein in Scutellaria root; [6]-shogaol, [8]-shogaol, [10]-shogaol, [6]-gingerol, [8]-gingerol, and [10]-gingerol in processed ginger, and glycyrrhetinic acid in Glycyrrhiza (Table 1). These results suggested that these ingredients could cooperatively enhance scratch-induced HOK migration.

In addition, we also confirmed that orally administered HST reduced the severity of OUM *in vivo* using a chemotherapy-administered OUM rat model. Consistent with this effect, topical HST (100 mg/mL) application significantly enhanced the healing of cutaneous wounds on day 14 following wound surgery (Supplementary Fig. S1). Notably, although oral HST administration increased body weight at day 6, at which time healing of OUM was enhanced (Fig. 5), the healing action of HST toward the cutaneous tissues was considered to be independent of body weight because of the equal effects of control (0 µg/mL) and HST (1, 10, 100 µg/mL)-administrated rats (Supplementary Fig. S1). These data suggested that the HST-evoked weight gain in the chemotherapy-administered OUM model might have been induced by an increase in food intake consequent to reduced OUM severity/OUM healing, which reduced the OUM-induced pain.

For the treatment of patients with cancer presenting with OUM, an important consideration would be to ensure that HST enhances neither the cell growth nor the migration of cancer cells. We found that, at the concentrations tested, HST did not increase and in some cases decreased the viability of various cancer cell lines including two derived from human squamous cell carcinoma of the tongue (HSC-4, SCC-25) human colorectal cancer (Fig. 6), nor did it increase HSC-4 and SCC-25 scratch-induced migration (Fig. 6). In addition, Miyashita T *et al*. have reported that HST reduced the incidence of reflux-induced esophageal cancer and the infiltration of macrophages in a surgical rat model^43^. Taken together, these findings indicated that HST does not promote the cancer itself, suggesting that HST may be useful for patients with cancer suffering from OUM. HST might not only ameliorate oral mucositis but also suppress growth of cancer cells, although further study is absolutely required.

In clinical practice for OUM treatment, HST was dissolved in hot water and administered by mouth-washing^22,44^. Therefore, HST may have two modes of action: directly affecting OUM, or affecting OUM following absorption in the blood. As shown in our plasma pharmacokinetic study, the doses of compounds derived from Scutellaria root, processed ginger, and Glycyrrhiza shown to be effective in the HST-induced HOK migration assay were higher than those found in the plasma (Fig. 3 and Table 1). In addition, our previous study has shown that the doses of components of the HST solution administered in clinical practice were 1–100 µM^26^. In comparison, the dose of baicalein required for HST-induced HOK migration was 10 µM and that of the ingredients of processed ginger was 0.1–10 µM (Table 1). Therefore, the doses in the HST-induced HOK migration were the same or lower than that of HST solution in clinical practice. Taken together, these finding suggested that the wound healing function afforded by HST is evoked by the direct action of HST on OUM.

Numerous groups have reported that MAPKs and CXCR4 play important roles in cell migration^29–42^. In our present study, HST enhanced the scratch-induced migration of HOK via MAPKs and CXCR4 (Fig. 4). Chao *et al*.^32^ and Su *et al*.^42^ have shown that baicalein activates JNK and/or p38. In addition, [6]-shogaol and [10]-gingerol activated ERK, JNK and p38^37,39,40^. These reports suggest that baicalein, [6]-shogaol and [10]-gingerol phosphorylate MAPKs and induce migration of HOKs. However, further study is needed to elucidate which ingredients activate MAPKs.

In addition, several reports have demonstrated relationships between MAPKs and CXCR4 in cell migration, which have been classified into two pathways: 1) MAPK activation increases the expression of CXCR4 and/or its ligands such as CXCL11 and CXCL12, and 2) MAPKs are involved in the cell migration induced by activation of CXCR4. With regard to the former, it has been reported that CXCR4 expression is induce d by ERK activation, with expression of CXCL-11 and CXCL-12, both of which are ligands for CXCR4, also being increased through activation of ERK and p38^31,33–35^. For the latter, some reports have shown that CXCL12 evokes T-cell migration through ERK activation, with the CXCR4-activated cell migration being associated with activation of ERK and/or p38^29,30,36,41^. It has also been reported that cell migration induced by the CXCL12/CXCR4 axis is due to the activation of JNK and p38^38^. To clarify the pathways underlying the beneficial effects of HST, studies investigating the role of HST and its constituents on the mRNA expression of CXCR4, CXCL11, and CXCL12 in HOK are now on-going in our laboratory.

Although chemotherapy-induced OUM is associated with the use of various anti-cancer drugs, there are not so many effective prevention methods or therapeutic modalities. HST thus constitutes a promising therapy, the efficacy of which has been demonstrated via a clinical study with a high evidence level^22,44^. Basic research findings have indicated that HST enhances OUM healing through multiple pharmacological actions (e.g., anti-oxidant, anti-inflammatory, anti-bacterial, and analgesic activities)^23–25^. With regard to anti-inflammatory effects, we have shown that various ingredients in HST decrease the interleukin 1β-induced prostaglandin E2 (PGE2) production in HOKs with multi-targeting effects, such as dual suppression of cyclooxygenase-2 expression and PGE2 metabolic activity^26^. For analgesic effects, the swab application of HST on an OUM rat model suppressed spontaneous and mechanical pain through Na^+^ channel blockage, which was mediated by several ingredients in HST^27,28^. We now add a new pharmacological action by demonstrating that HST exhibits wound healing activity, with its pharmacological actions being regulated by different the constituent medicinal herbs (Supplementary Table 1). Taken together, these data suggested that the seven medicinal herbs comprising HST exert different pharmacological actions in the treatment of cancer therapy-induced OUM.

Irinotecan, an anti-cancer drug, exerts an inhibitory effect on nucleic acid synthesis by inhibition of topoisomerase I^45^. One of the dose-limiting toxicities is diarrhea (beginning more than 24 h after infusion) that leads to dehydration and electrolyte imbalance, making it a life-threatening condition^46^. The mechanism of irinotecan-induced diarrhea is direct damage to the intestinal mucosa induced by SN-38, which is a metabolite and an active form of irinotecan^47^. In an animal experiment, HST exhibited protective effects against intestinal toxicity caused by irinotecan^48^. In addition, a randomized controlled trial had revealed that the grade of diarrhea significantly improved after treatment with HST in patients with non-small cell lung cancer treated with cisplatin and irinotecan^49^. HST significantly reduced the frequency of severe diarrhea (grade 3 or 4). Although further studies is needed, one of the mechanism of HST on the improvement of irinotecan-induced diarrhea could be HST-induced migration of intestinal epithelial cells.

In conclusion, the findings of the present study suggested that HST, specifically the active ingredients in its constituents Scutellaria root, Processed ginger, and Glycyrrhiza, has direct beneficial effects on OUM and enhances tissue repair through oral keratinocyte migration as likely induced by activation of MAPKs and CXCR4. This study provides scientific evidence supporting the use of HST in patients with chemotherapy-induced OUM. Further studies should be performed on the efficacy, safety and mechanisms of this promising agent for the management of mucositis in cancer patients treated with other treatment modalities.

## Methods

### Chemicals and reagents

The following reagents were used: foetal bovine serum (FBS) and Keratinocyte-SFM (1 × ) (Gibco, Carlsbad, CA, USA); trypsin and trypsin neutralizing solution (TNS) (Lonza, Basel, Switzerland); penicillin/streptomycin, dimethyl sulphoxide (DMSO), U0126 (Nacalai Tesque, Kyoto, Japan); poly-L-lysine (PLL), SB202190, 5-fluorouracil and glycyrrhetinic acid (Sigma-Aldrich, St. Louis, MO, USA); Cellmatrix I-P (Nitta Gelatin Inc., Osaka, Japan); phosphate buffered saline (–) (PBS); (Nissui Pharmaceutical Co., Osaka, Japan); Dulbecco’s modified Eagle medium (DMEM) Low Glucose, RPMI-1640, baicalin, wogonin, berberine chloride, rutin, and BDPA-Zn (Fujifilm Wako Pure Chemical, Osaka, Japan); JNK inhibitor II (Calbiochem, San Diego, CA, USA); medetomidine (zenoaq, Fukushima, Japan), midazolam (Sandoz, Yamagata, Japan); butorphanol (Meiji Seika Pharma, Tokyo, Japan); and thalifendine chloride and demethyleneberberine chrolide (Cheminstock, Shanghai, China).

HST extract powder (Lot No. 2100014010), the base powder of HST without excipients, was obtained from Tsumura & Co. (Ibaraki, Japan), manufactured as an aqueous extract mixture of seven medicinal herbs (percentage): Pinellia tuber (27.0), Scutellaria root (13.5), processed ginger (13.5), Glycyrrhiza (13.5), jujube (13.5), ginseng (13.5), and Coptis rhizome (5.5). The dried powdered extract form of HST and its crude drugs were suspended in DMSO at 100 mg/mL, diluted 100 fold with culture medium, and filtered through a 0.45 μm membrane (ADVANTEC, Tokyo, Japan), then applied to cells at 1, 10, 30, or 100 μg/mL (final concentration).

Among HST ingredients, baicalein, wogonoside, [6]-shogaol, [8]-shogaol, [10]-shogaol, [6]-gingerol, [8]-gingerol, [10]-gingerol, coptisine, berberrubine, jatrorrhizine, isoliquiritigenin, ginsenoside Rb1, 6-C-xylopyranosyl-8-C-galactopyranosylapigenin (XGA), and 6-C-galactopyranosyl-8-C-xylopyranosylapigenin (GXA), with purities sufficiently high for evaluation in biological tests, were used (Tsumura & Co.) All other reagents were of the highest purity available from commercial sources.

### Cell culture

Primary human oral keratinocytes (HOKs, Lot No. 5854, ScienCell Research Laboratories, Carlsbad, CA, USA) were cultured on PLL-coated dishes in Keratinocyte-SFM (1×) supplemented with 10% FBS, penicillin (100 U/mL).

In cancer cells, human squamous cell carcinoma cells of the tongue (HSC-4, SCC-25), colorectal cancer cells (DLD-1), and human gastric cancer cells (MKN-45) were used in this study. HSC-4 and SCC-25 cells were cultured in DMEM Low Glucose supplemented with 10% FBS, penicillin (100 U/mL), and streptomycin (100 mg/mL). DLD-1 and MKN-45 cells were cultured in RPMI-1640 supplemented with 10% FBS, penicillin (100 U/mL), and streptomycin (100 mg/mL). All cells were incubated at 37 °C in a humidified atmosphere of 95% air and 5% CO_2_.

### Scratch-induced migration assay

HST effects on scratch-induced migration of HOK or cancer cells were evaluated using the IncuCyte ZOOM system (ESSEN BioScience, Ann Arbor, MI, USA), which enables real-time and quantitative live-cell analysis. These cells were seeded at 3.0 × 10^4^ (HOK), 2.5 × 10^4^ (HSC-4) or 1.5 × 10^4^ (SCC-25) cells/well in 96 well ImageLock microplates (ESSEN BioScience) coated with 300 µg/mL collagen (Cellmatrix^®^ I-P), respectively. The next day, cells were scratched using a 96 well WoundMaker (ESSEN BioScience) and the culture medium was changed to assay medium [Keratinocyte-SFM (1×) containing 2% FBS (HOK), DMEM Low Glucose (HSC-4 and SCC-25) or RPMI-1640 (DLD-1 and MKN-45)]. Cells were treated with HST, medicinal herbs, or various ingredients and visually monitored every 2 h for up to 72 h. The area occupied by cells on the scratched area was quantified using IncuCyte scratch wound cell migration software (ESSEN BioScience).

### Cell viability assay

Cell viability assay was performed using Cell Counting Kit-8 according to manufacturer instruction (Dojindo Laboratories, Kumamoto, Japan). Briefly, cells were seeded at 1.5 × 10^4^ cells/well (HOK), 3 × 10^4^ cells/well (HSC-4) or 7 × 10^4^ cells/well (DLD-1, MKN-25, and SCC-25) in 96-well microplates (Corning, Armonk, NY, USA) and incubated overnight. The culture medium was changed to assay medium [Keratinocyte-SFM (1×) containing 2% FBS (HOK), DMEM Low Glucose (cancer cells)] and the cells were treated with HST. After incubation at 37 °C for 72 h, Cell Counting Kit-8 reagents were applied and the absorbance measured at 450 nm.

### Experimental animals for plasma pharmacokinetic analysis of HST-derived ingredients

Male Sprague-Dawley rats aged 6 weeks (Japan SLC, Shizuoka, Japan) were used after one-week habituation. The animals were allowed free access to water and standard laboratory food, and housed at 20–26 °C, 30–70% relative humidity, and 12 h light:12 h dark cycle (07:00 to 19:00 lights on). All experimental procedures were performed according to the Tsumura & Co. ‘Guidelines for the Core and Use of Laboratory Animals’ and approved by the Tsumura & Co. Laboratory Animal Committee for the ethical and experimental procedures.

### Drug administration and plasma collection

HST extract powder (0.9–1.1 g/kg) dissolved in 10 mL distilled water was orally administered to 16 h fasted rats. Animals (*n* = 3/each point) were sacrificed at 0 (pre-administration), 0.25, 0.5, 1, 2, 4, 8, 24, 32, and 48 h following administration by exsanguination from the abdominal postcava. Plasma samples were collected from the blood by centrifugation (1,700 *g*, 15 min, 4 °C) and stored at −80 °C until analysis.

### Plasma pharmacokinetic analysis of HST-derived ingredients

For pharmacokinetic analysis, we focused on the main ingredients in each crude HST component and their metabolites (Table 2). To quantify the compounds, the plasma samples were analysed using liquid chromatography-tandem mass spectrometry (LC-MS/MS) following pretreatment as described below. The LC-MS/MS system consisted of an API4000 triple quadrupole mass spectrometer (SCIEX, Framingham, MA, USA) equipped with an Agilent 1100 system (Santa Clara, CA, USA).

For glycyrrhetinic acid and isoliquiritigenin quantification, 200 µL plasma was mixed with 25 µL acetonitrile, an equal volume of niflumic acid (100 ng/mL) as an internal standard, and 750 µL methanol. To prepare the calibration curve, the same volumes of various concentrations of working solution were used instead of the acetonitrile. The mixture was centrifuged (3,000 *g*, 4 °C, 15 min), then 750 µL supernatant was dried under a stream of nitrogen gas and dissolved in 50 µL of a solvent such as the initial mobile phase used for the LC-MS/MS systems.

For baicalin, baicalein wogonin, wogonoside, [6]-shogaol, [8]-shogaol, [10]-shogaol, [6]-gingerol, [8]-gingerol, and [10]-gingerol quantification, 200 µL plasma was mixed with 25 µL acetonitrile or various concentrations of working solution, an equal volume of niflumic acid (100 ng/mL), and 150 µL water. The mixture was centrifuged (1,700 *g*, 4 °C, 2 min), then the total supernatant volume was loaded onto a preconditioned (1 mL methanol and 1 mL water) Oasis HLB cartridge (Waters, Milford, MA, USA). The cartridge was washed with water, then analytes were eluted with 400 µL methanol and/or 400 µL acetonitrile, dried under nitrogen gas, and dissolved in 50 µL of a solvent as described above. LC-MS/MS analytical conditions are summarized in Supplementary Tables 2 and 3.

### Experimental animals for the OUM model

Male Wistar rats aged 8 weeks (Kyudo, Saga, Japan, *n* = 14) were housed in pairs in clear cages with wood chips under specific-pathogen-free conditions, and maintained on a 12:12 h light-dark cycle in a temperature- and humidity-controlled room (21–23 °C and 40–60%, respectively) with food pellets and water provided *ad libitum*, except during the experimental period. All *in vivo* experiments were conducted in accordance with the National Institutes of Health Guide for the Care and Use of Laboratory Animals and approved by the Animal Experiment Committee of Kyushu Dental University (approval nos. 18–014 and 18–025). Rats were randomly selected for each experiment. Evaluation of OUM severity and cutaneous wound healing was performed in a manner blind to the experimental conditions.

### Generation of chemotherapy-administered OUM

According to our previous study^50^, the representative chemotherapy drug 5-fluorouracil (40 mg/kg/day in saline) was intraperitoneally administered 3 times at 2-day intervals in rats. Two days after the final administration, the labial fornix region of the inferior incisors was treated with a filter paper (3 mm × 3 mm, Whatman, Maidstone, UK) soaked in 50% acetic acid soaked for 30 s under anaesthesia (mixture of medetomidine (0.375 mg/kg), midazolam (2 mg/kg), and butorphanol (2.5 mg/kg) by intraperitoneal administration.

### HST application and OUM healing analysis

For acclimation, rats were fed a powder diet instead of pellets from 1 week prior to the OUM healing experiments. From the day of acetic acid treatment, rats were fed the powder diet containing 1% HST or normal powder diet (control) until the end of the experiment.

To evaluate OUM severity, we used the following visual oral mucositis score, modified from that for a radiation-induced OUM model^50,51^: 0, normal; 0.5, possible presence of redness; 1, slight but definite redness; 2, severe redness; 3, focal pseudomembrane, without a break in the epithelium; 4, broad pseudomembrane, with a break in the epithelium within the acetic acid-treated mucosal area; 5, virtual loss of epithelial and keratinized layers over the acetic acid-treated mucosal area; and 6, severe swelling of the lower lip with OUM at a score of 5. The evaluation was performed every day under 2% isoflurane anaesthesia.

### Statistical analysis

The data are presented as the means ± S.E.M. Statistical analysis was performed using one- or two-way analysis of variance (ANOVA) followed by Tukey’s or Sidak’s multiple comparisons tests (GraphPad Prism 6, San Diego, CA, USA). p < 0.05 was considered statistically significant.

## Supplementary information

Supplementary information

Supplementary information2

Supplementary information3
