# Author Correction: The Japanese herbal medicine Hangeshashinto enhances oral keratinocyte migration to facilitate healing of chemotherapy-induced oral ulcerative mucositis

**DOI:** 10.1038/s41598-020-76581-6

**Published:** 2020-11-09

**Authors:** Kanako Miyano, Moeko Eto, Suzuro Hitomi, Takashi Matsumoto, Seiya Hasegawa, Ayane Hirano, Kaori Nagabuchi, Noriho Asai, Miaki Uzu, Miki Nonaka, Yuji Omiya, Atsushi Kaneko, Kentaro Ono, Hideaki Fujii, Yoshikazu Higami, Toru Kono, Yasuhito Uezono

**Affiliations:** 1 Division of Cancer Pathophysiology, National Cancer Center Research Institute, Tokyo, 104-0045 Japan; 2 Laboratory of Molecular Pathology and Metabolic Disease, Faculty of Pharmaceutical Sciences, Tokyo University of Science, Chiba, 278-8510 Japan; 3 Division of Physiology, Kyushu Dental University, Fukuoka, 803-8580 Japan; 4 Tsumura Kampo Research Laboratories, Tsumura & Co., Ibaraki, 300-1192 Japan; 5 Department of Medicinal Chemistry, School of Pharmacy, Kitasato University, Tokyo, 108-8641 Japan; 6 Translational Research Center, Research Institute of Science and Technology, Tokyo University of Science, Chiba, 278-8510 Japan; 7 Center for Clinical and Biomedical Research, Sapporo Higashi Tokushukai Hospital, Sapporo, Hokkaido Japan; 8 Division of Supportive Care Research, Exploratory Oncology Research and Clinical Trial Center, National Cancer Center, Tokyo, 104-0045 Japan; 9 Innovation Center for Supportive, Palliative and Psychosocial Care, National Cancer Center Hospital, Tokyo, 104-0045 Japan

Correction to: *Scientific Reports* 10.1038/s41598-019-57192-2, published online 17 January 2020

This Article contains errors where n values were omitted from some of the Figure and Table legends.

In the legend of Figure 1,

“(**b**) Effect of HST (1, 10, 100 μg/mL) on the scratch-induced migration of HOKs over time. (**c**) Extent of the scratch-induced migration of HOKs treated with HST (1, 10, 100 μg/mL) for 72 h calculated as the ratio of vehicle [confluence of HOKs (%) in wound area treated with HST/that treated with vehicle]. (**d**) Cell viability in HOKs treated with vehicle or HST (1, 10, 100 μg/mL) for 72 h. Data are expressed as the ratio of vehicle at 72 h.”

should read:

“(**b**) Effects of HST (1, 10, 100 µg/mL) on the scratch-induced migration of HOKs over time (n = 12–13). (**c**) Extent of the scratch-induced migration of HOKs treated with HST (1, 10, 100 µg/mL) for 72 h calculated as the ratio of vehicle [confluence of HOKs (%) in wound area treated with HST/ that treated with vehicle (n = 12–13). (**d**) Cell viability in HOKs treated with vehicle or HST (1, 10, 100 µg/mL) for 72 h (n = 14). Data are expressed as the ratio of vehicle at 72 h.”

In the legend of Figure 2,

“HOKs were treated with 1, 10, or 30 μg/mL of Pinellia tuber (**a**), Scutellaria root (**b**), processed ginger (**c**), Glycyrrhiza (**d**), jujube (**e**), ginseng (**f**), or Coptis rhizome (**g**).”

should read:

“HOKs were treated with 1, 10 or 30 µg/mL of pinellia tuber (**a**), Scutellaria root (**b**), processed ginger (**c**), Glycyrrhiza (**d**), jujube (**e**), ginseng (**f**), and Coptis rhizome (**g**) (n = 15–18)."

In the legend of Table 1,

“HOKs were treated with various ingredients of Scutellaria root (baicalin, baicalein, and wogonin), processed ginger ([6]-shogaol, [8]-shogaol, [10]-shogaol, [6]-gingerol, [8]-gingerol, and [10]-gingerol), and Gglycyrrhiza (glycyrrhizin, liquiritin, isoliquiritin, liquiritin apioside, and isoliquiritigenin) for 72 h. Data are expressed as the ratio of vehicle at 72 h.”

should read:

“HOKs were treated with various ingredients of scutellaria root (baicalin, baicalein and wogonin), processed ginger ([6]-shogaol, [8]-shogaol, [10]-shogaol, [6]-gingerol, [8]-gingerol and [10]-gingerol) and Glycyrrhiza (glycyrrhizin, Liquiritin, Isoliquiritin, Liquiritin apioside, and Isoliquiritigenin) for 72 h (n = 11–20). Data are expressed as the ratio of vehicle at 72 h.”

In the legend of Figure 4,

“HOKs were co-treated with HST (100 μg/mL) and an ERK inhibitor U0126 (10 μM, **a**), JNK inhibitor II (1 μM, **b**), p38 inhibitor SB202190 (10 μM, **c**), or a CXCR4 inhibitor BDPA-Zn (3 μM, **d**).”

should read:

“HOKs were co-treated with HST (100 µg/mL) and an ERK inhibitor U0126 (10 µM, **a**), a JNK inhibitor II (1 µM, **b**), a p38 inhibitor SB202190 (10 µM, **c**) or a CXCR4 inhibitor BDPA-Zn (3 µM, **d**) (n = 18–51)."

In the legend of Figure 6,

“HSC-4 (**a**), SCC-25 (**b**), DLD-1 (**c**), and MKN-45 (**d**) cell lines were treated with HST for 72 h, then cell growth was measured using Cell Counting Kit-8. HSC-4 (**e**) and SCC-25 (**f**) cells were treated with HST (1, 10, and 100 μg/mL) for 72 h, then the area occupied by cancer cells on the scratched area was quantified using IncyCyte scratch wound cell migration software (ESSEN BioScience).”

should read:

“HSC-4 (**a**), SCC-25 (**b**), DLD-1 (**c**) and MKN-45 (**d**) cell lines were treated with HST for 72 h, then cell growth were measured using Cell Counting Kit-8 (n = 9). HSC-4 (**e**, n = 8–16) and SCC-25 (**f**, n = 3–7) cells were treated with HST (1, 10, 100 µg/mL) for 72 h, then the area occupied by cancer cells on the scratched area were quantified using IncuCyte scratch wound cell migration software (ESSEN BioScience).”

