# Supplementary material for: The Japanese herbal medicine Hangeshashinto enhances oral keratinocyte migration to facilitate healing of chemotherapy-induced oral ulcerative mucositis
Source: Sci Rep. 2020 Jan 17;10:625. doi: 10.1038/s41598-019-57192-2 (PMC6969174; doi:10.1038/s41598-019-57192-2)
Supplement: Supplementary file 3 — Supplementary information3 [file 41598_2019_57192_MOESM3_ESM.docx]

**Supplementary information**

**The Japanese herbal medicine Hangeshashinto enhances oral keratinocyte migration to facilitate healing of chemotherapy-induced oral ulcerative mucositis**

Kanako Miyano, Moeko Eto, Suzuro Hitomi, Takashi Matsumoto, Seiya Hasegawa, Ayane Hirano, Kaori Nagabuchi, Noriho Asai, Miaki Uzu, Miki Nonaka, Yuji Omiya, Atsushi Kaneko, Kentaro Ono, Hideaki Fujii, Yoshikazu Higami, Toru Kono & Yasuhito Uezono^*^

*Correspondence and requests for materials should be addressed to Y. U. (email: yuezono@ncc.go.jp)

**Supplementary Methods**

**Cutaneous wound-making**. Male Wistar rats aged 14-17 weeks (Kyudo, Saga, Japan, n = 9) were used in cutaneous wound model. Under anaesthesia by intraperitoneal administration of a mixture of medetomidine (0.375 mg/kg), midazolam (2 mg/kg), and butorphanol (2.5 mg/kg), the dorsal surface of rats was de-haired by shaving with an electric clipper followed by treatment with a depilatory agent (Epilat, Kracie, Tokyo, Japan) to remove any remaining hair. After being rinsed with saline and sterilized with betadine, 8 mm full-thickness excisional skin wounds were created on the dorsal surfaces (two wounds on the left and right sides) via a sterile disposable punch biopsy (Kai Medical, Solingen, Germany). Following the surgery, the rats were housed in individual cages to exclude the risk of other rats chewing the wound.

**HST treatment and wound analysis**. Would generation is described in Supplementary Methods. HST at 0, 1, 10, and 100 mg/mL (HST-0, HST-1, HST-10, and HST-100, respectively) in distilled water was applied to the wounds with the hydrofibre dressing (Aquacell; Convatec, Skillman, NJ, USA), then wounds were covered by film dressings (KYOWA, Osaka, Japan). The drug solutions with hydrofibre dressing were re-applied at 2 day intervals under 2% isoflurane anaesthesia. For analysis, each wound region was traced to tracing paper and the lengths of major- and minor axes were measured. The wound area was calculated via the lengths.

**Results**

**Effect of HST on cutaneous wound healing in rats.** The cutaneous wound areas treated with 1, 10, and 100 µg/mL HST (HST-1, HST-10, and HST-100, respectively) were smaller than those treated with 0 µg/mL HST (HST-0), but not significantly, on day 7 after the wound surgery (*n* = 8–9, Supplementary Fig. 1a and c). On day 14, the wound area in HST-100 was significantly smaller than that in HST-0 (Supplementary Fig. S1d).

**
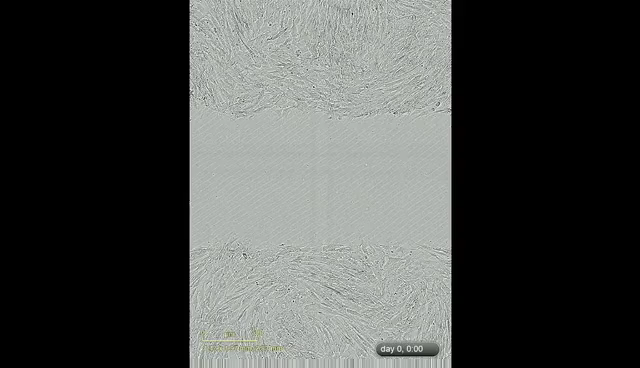
**

Vehicle (vehicle.mp4)

**
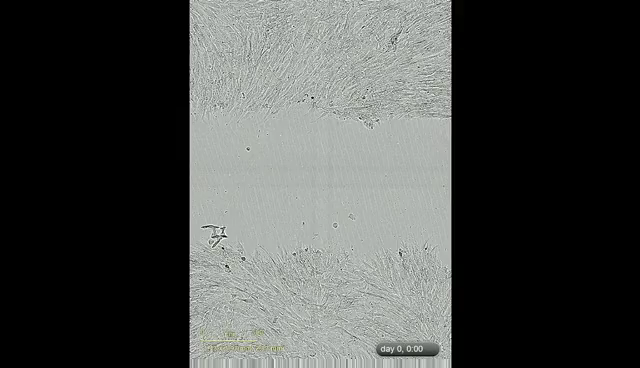
**

HST 100 μg/mL

(HST 100ug/mL.mp4)

**Supplementary Movie S1. Movies of the scratch-induced wound healing using HOKs.**  Scratch-induced migration was measured using the IncuCyte^TM^ ZOOM system every 2 h for up to 72 h. Movies were generated by connecting these photographs, which show the image of the scratched area in HOKs treated with vehicle (**a**) or 100 µg/mL HST (**b**).


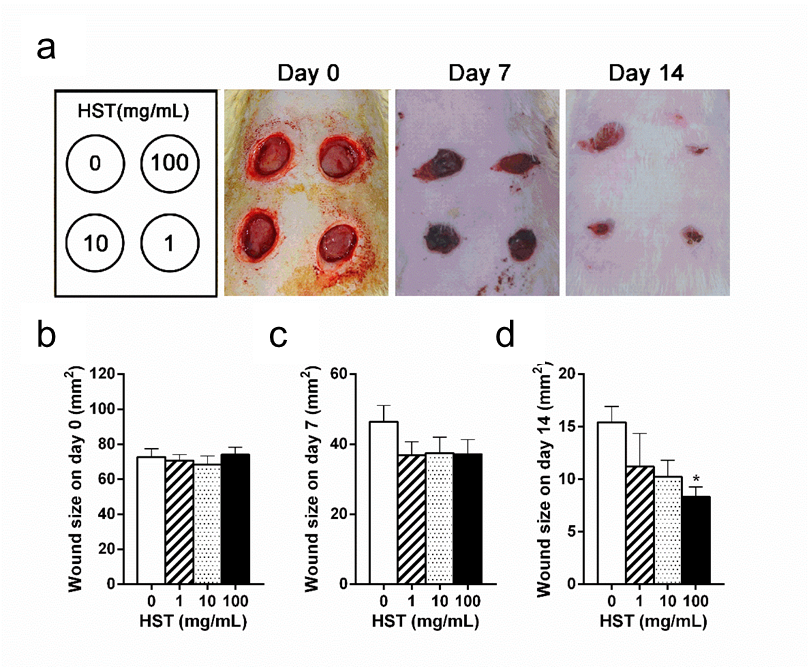


**Supplementary Figure S1. Effect of HST on cutaneous wound healing in rats.** (**a**) Representative photographs of the wound area on days 0, 7, and 14 following wound surgery. The left schema indicates the drug concentration of HST applied to each wound. (**b**-**d**) Change of wound size on days 0, 7, and 14 following wound surgery. *n* = 8–9. * Indicates p < 0.05 compared with 0 mg/mL; Dunnett’s multiple comparisons test following one-way ANOVA.

**Supplementary Table 1. Regulation of the pharmacological activity by the various components of HST**

**
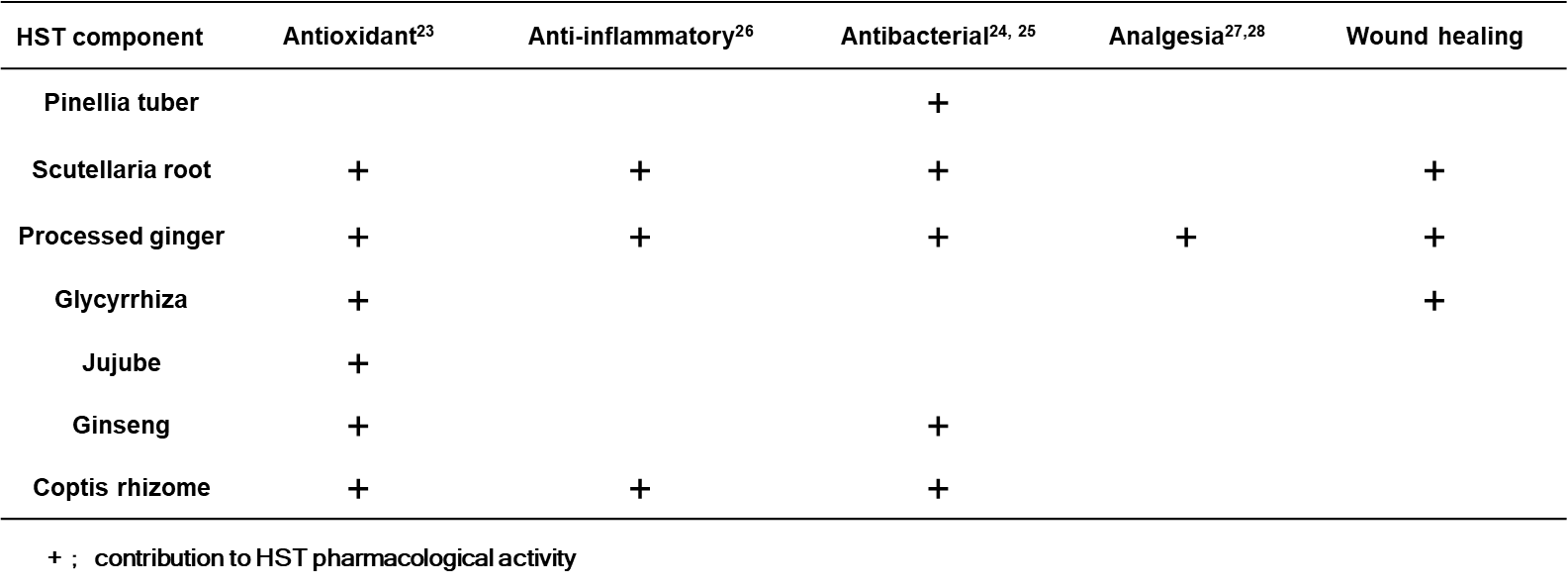
**

**Supplementary Table 2. Methods of LC-MS/MS: Ion parameters of HST components.**

| Component | Q1 (*m/z*) | Q3 (*m/z*) | DP (volts) | CE (volts) | CXP (volts) | # |
| --- | --- | --- | --- | --- | --- | --- |
| Baicalin | 445.0 | 269.0 | −75 | −24 | −17 | 1 |
| Baicalein | 269.0 | 138.9 | −95 | −44 | −25 | 1 |
| Wogonin | 282.9 | 268.0 | −65 | −24 | −23 | 1 |
| Wogonoside | 459.1 | 282.9 | −70 | −24 | −19 | 1 |
| [6]-gingerol | 295.2 | 137.1 | 41 | 33 | 24 | 2 |
| [8]-gingerol | 323.2 | 305.3 | 56 | 7 | 16 | 2 |
| [10]-gingerol | 351.2 | 137.2 | 36 | 31 | 8 | 2 |
| [6]-shogaol | 276.5 | 137.2 | 21 | 35 | 10 | 2 |
| [8]-shogaol | 305.2 | 137.2 | 76 | 15 | 12 | 2 |
| [10]-shogaol | 333.3 | 137.1 | 64 | 19 | 24 | 2 |
| Glycyrrhetinic acid | 469.4 | 355.4 | −155 | −62 | −19 | 3 |
| Isoliquiritigenin | 254.9 | 118.9 | −75 | −36 | −19 | 3 |
| Niflumic acid (IS) | 280.8 | 236.8 | −55 | −24 | −11 | 1, 3 |
| Niflumic acid (IS) | 283.1 | 265.1 | 101 | 31 | 20 | 2 |

#: HPLC method IDs are described in Supplementary Table 3.

DP; declustering potential, CE; collision energy, CXP; collision cell exit potential, IS; internal standard.

**Supplementary Table 3. Details of LC-MS/MS:HPLC method conditions.**

| HPLC method | HPLC condition |
| --- | --- |
|  |  |
| 1 | Column: YMC-Pack ODS-AQ column (50 × 2.0 mm I.D., 3 μm particle size; YMC Co., Kyoto, Japan) |
|  | Mobile phase (A) 0.2 vol % formic acid, (B) methanol containing 0.2 vol % formic acid |
|  | Gradient elution program (% B in A): |
|  | 0–8 min, 50–100%; 8–10 min, 100%; 10.01–13 min, 50% |
|  | Other conditions were: flow rate, 0.2 mL/min; column temperature, 40 °C |
| 2 | Column: YMC-Pack ODS-AQ column (50 × 2.0 mm I.D., 3 μm particle size; YMC Co.) |
|  | Mobile phase (A) 0.2 vol % acetic acid, (B) acetonitrile containing 0.2 vol % acetic acid |
|  | Gradient elution program (% B in A): |
|  | 0–10 min, 15–90%; 10–15 min, 90%; 15.01–19 min, 15% |
|  | Other conditions were: flow rate, 0.2 mL/min; column temperature, 40 °C |
| 3 | Column: Inertsil Ph-3 column (100 × 2.1 mm I.D., 3 μm particle size; GL Sciences, Tokyo, Japan) |
|  | Mobile phase (A) 0.2 vol % formic acid, (B) methanol containing 0.2 vol % formic acid |
|  | Gradient elution program (% B in A): |
|  | 0–4 min, 2%; 4.01–10 min, 32%; 10–10.01 min, 32–95%; 13–18 min; 100%; 18.01–23 min, 2% |
|  | Other conditions were: flow rate, 0.3 mL/min; column temperature, 40 °C |
